# Polyclonal distribution and predicted plasmid context of ESBL- and AmpC-associated resistance in chicken-derived *Escherichia coli* from Hungary

**DOI:** 10.3389/fvets.2026.1959409

**Published:** 2026-10-02

**Authors:** Ádám Kerek, Gergely Álmos Tornyos, Levente Hunor Husz, Máté Hetyésy, Krisztina Bali, Levente Molnár, Szonja Petra Kiss, Ákos Jerzsele, Eszter Kaszab

**Affiliations:** 1 Department of Pharmacology and Toxicology, University of Veterinary Medicine Budapest, Budapest, Hungary; 2 National Laboratory of Infectious Animal Diseases, Antimicrobial Resistance, Veterinary Public Health and Food Chain Safety, University of Veterinary Medicine Budapest, Budapest, Hungary; 3 Department of Microbiology and Infectious Diseases, University of Veterinary Medicine Budapest, Budapest, Hungary; 4 Department of Bioinformatics, One Health Institute, Faculty of Health Sciences, University of Debrecen, Debrecen, Hungary

**Keywords:** AmpC, antimicrobial resistance, *Escherichia coli*, extended-spectrum β-lactamase, plasmids, poultry

## Abstract

**Background:**

Poultry-associated *Escherichia coli* can harbor clinically relevant antimicrobial resistance determinants, but the clonal and mobile-genetic backgrounds of β-lactam resistance remain incompletely characterized in Central European production systems. We investigated a phenotypically ESBL-suspect collection of 87 *E. coli* isolates recovered from tracheal and cloacal samples collected on 23 commercial chicken farms in Hungary between 2022 and 2023.

**Methods:**

Antimicrobial susceptibility testing was integrated with whole-genome sequencing, allele-level resistance-gene identification, Achtman multilocus sequence typing, predicted plasmid reconstruction, pan-genome analysis and recombination-filtered core-genome phylogenetics.

**Results:**

Phenotypic ESBL production was confirmed in 19/87 isolates (21.8%). The principal ESBL alleles were *bla*_CTX-M-1_ (11/87) and *bla*_CTX-M-15_ (3/87), while *bla*_CMY-2_ was detected in four isolates. Complete Achtman sequence types were assigned to 44 isolates, representing 30 sequence types. Ten of 11 *bla*_CTX-M-1_ occurrences were computationally assigned by MOB-suite to predicted conjugative IncI-gamma/K1 plasmid bins. The recombination-filtered phylogeny resolved multiple distinct lineages, showing that ESBL positivity and CTX-M carriage were not restricted to a single clone.

**Conclusion:**

These findings indicate that ESBL- and AmpC-associated resistance occurred across diverse chicken-derived *E. coli* backgrounds and was frequently associated with predicted plasmid contexts.

## 1 Introduction

Antimicrobial resistance (AMR) is a major threat to the sustained effectiveness of antibacterial therapy in both human and veterinary medicine. Food-producing animals may harbor antimicrobial-resistant commensal bacteria and mobile resistance determinants, creating opportunities for their persistence and circulation within production environments and along the food chain. Surveillance of resistance in indicator organisms such as *Escherichia coli* is therefore important for identifying emerging resistance mechanisms and understanding the bacterial and mobile-genetic backgrounds in which they occur (1).

Among Gram-negative pathogens, *E. coli*—a facultative commensal of the intestinal microbiota—holds particular importance. While typically harmless, certain pathogenic strains can cause severe disease, especially in immunocompromised hosts. In poultry, pathogenic *E. coli* strains are associated with respiratory infections, septicemia, and substantial economic losses (2). In young chicks, septicemia is common, whereas in adult birds respiratory infections and organ-specific localizations—such as airsacculitis, peritonitis, and coligranulomatosis—are more frequent (3). Beyond its role as a primary pathogen, *E. coli* also serves as a major reservoir of antimicrobial resistance genes, facilitating their dissemination across ecological and host boundaries.

A critical contributor to AMR is the global spread of *E. coli* strains producing extended-spectrum β-lactamases (ESBLs), enzymes that hydrolyze a broad range of cephalosporin antibiotics (4). These bacteria exhibit resistance to multiple β-lactam antibiotics—including penicillins, cephalosporins, and monobactams—while often retaining susceptibility to carbapenems and β-lactamase inhibitors (5). The widespread emergence of ESBL-producing *E. coli* poses a substantial challenge not only to clinical therapy, but also to infection control and surveillance across veterinary and human health sectors.

Historically, *bla*_TEM_ and *bla*_SHV_-type ESBL genes were predominantly detected in hospital-acquired *E. coli* infections. However, over the past two decades, *bla*_CTX-M_-type ESBLs have become increasingly prevalent across continents (6). The extensive use of β-lactam antibiotics in food-producing animals has facilitated the selection and dissemination of plasmid-encoded ESBL genes within animal-derived *E. coli* strains, now detected in swine, cattle, and poultry globally (7). The plasmid-mediated nature of these resistance determinants highlights the importance of horizontal gene transfer in shaping the epidemiology of ESBL-producing bacteria.

The magnitude of resistance in *E. coli* populations is concerning (8). A study conducted in the Netherlands (2011–2012) reported ESBL-producing *E. coli* in 65% of fecal samples from laying hen farms and 81% in broiler flocks. These resistant strains were also isolated from environmental reservoirs such as wastewater, sediments, farm dust, surface waters, and even from flies and air samples (9). Such findings underscore the role of poultry production systems as amplification points for antimicrobial-resistant bacteria with potential for environmental dissemination and zoonotic transmission.

Whole-genome sequencing enables the simultaneous characterization of antimicrobial resistance determinants, bacterial lineages and mobile-genetic contexts (10). Multilocus sequence typing (MLST) provides a standardized framework for comparing bacterial lineages by assigning a sequence type (ST) to a defined combination of alleles at housekeeping loci. The Achtman scheme for *E. coli* comprises seven loci: *adk*, *fumC*, *gyrB*, *icd*, *mdh*, *purA* and *recA*. In poultry-associated *E. coli*, this approach can distinguish between clonal expansion of a successful resistant lineage and the repeated acquisition of resistance determinants by unrelated genomic backgrounds (11). Although chicken-only datasets cannot directly demonstrate transmission between animal and human populations, they provide an important sector-specific component of integrated antimicrobial-resistance surveillance (12).

Despite increasing interest in stewardship and non-antibiotic interventions, effective mitigation of antimicrobial resistance still depends on integrated surveillance systems capable of identifying emerging resistance patterns in food-producing animals (13). In phenotypic surveillance, epidemiological cut-off values complement clinical breakpoints by distinguishing wild-type from non-wild-type MIC distributions (14).

In this study, we investigated an isolate-based, phenotypically ESBL-suspect collection of chicken-derived *E. coli* from commercial farms in Hungary. We hypothesized that ESBL-associated resistance would be distributed across multiple *E. coli* sequence types and predicted plasmid backgrounds rather than being attributable to the expansion of a single dominant clone. To test this hypothesis, we integrated antimicrobial susceptibility testing with allele-level β-lactamase identification, Achtman multilocus sequence typing, predicted plasmid reconstruction, virulence-gene screening, pan-genome analysis and recombination-filtered core-genome phylogenetics.

## 2 Materials and methods

### 2.1 Sample collection and bacterial isolation

This observational, cross-sectional study aimed to characterize a β-lactam/ESBL-oriented collection of *E. coli* isolates recovered from clinically healthy chickens under commercial production conditions. Sample collection was conducted between 2022 and 2023 on 23 large-scale commercial chicken farms in Hungary, encompassing broiler, laying and breeding production systems.

Each specimen was labeled with a unique identifier, and the anatomical source—either trachea or cloaca—was recorded. At each farm, the same 15 chickens were sampled at both anatomical sites. One tracheal and one cloacal swab were collected from each bird, yielding 30 paired specimens per farm and 690 specimens overall. A fixed sampling effort of 15 birds per farm was applied consistently across all 23 farms. No *a priori* power calculation was performed because the study was designed for exploratory isolate characterization rather than for inference on within-bird differences between anatomical sites or for estimation of flock-level prevalence. Swabs were collected using Amies-type (carbon-free) aluminum-shaft swabs (Biolab Zrt., Budapest, Hungary). Sampling procedures were non-invasive and were performed as part of routine veterinary monitoring. The Animal Welfare Committee of the University of Veterinary Medicine Budapest formally confirmed that the study did not constitute an animal experiment requiring authorization under the applicable Hungarian legislation and issued an exemption certificate. As this document is an exemption certificate rather than an authorization decision, no protocol or approval number is assigned to it.

The samples were plated on ChromoBio Coliform agar (Biolab Zrt., Budapest, Hungary) for preliminary *E. coli* isolation. Colonies suspected of being *E. coli* were subcultured on tryptone soya agar (Biolab Zrt., Budapest, Hungary) and incubated for 18–24 h at 41 °C. Species identification was performed using MALDI-TOF MS (Flextra-LAB Ltd., Budapest, Hungary) with Biotyper software version 12.0 (Bruker Daltonics GmbH, Bremen, Germany, 2024) (15). Non-selective chromogenic coliform agar was used for primary isolation to avoid restricting bacterial recovery exclusively to cefotaxime-resistant or presumptive ESBL-producing subpopulations. Enrichment for β-lactam non-susceptibility was applied subsequently at the isolate-selection stage rather than during primary culture.

From each culture-positive specimen, a single MALDI-TOF MS-confirmed *E. coli* isolate was retained. The sole criterion for inclusion in the whole-genome sequencing set was an initial phenotypic antimicrobial-susceptibility profile suggestive of potential ESBL production. No additional selection criteria based on farm, production type, anatomical source or geographic region were applied. Phenotypic ESBL status was subsequently determined using the predefined cefotaxime–clavulanic acid confirmatory criterion described in Section 2.3. Consequently, isolates selected as potential ESBL producers during the initial phenotypic screening could subsequently be classified as either ESBL-positive or ESBL-negative by the confirmatory test. The resulting 87-isolate genomic collection therefore represents a phenotypically ESBL-suspect analytical set and was not designed to estimate sample-level, farm-level or flock-level prevalence.

The confirmed *E. coli* isolates were stored at −80 °C in Micro-bank cryovials (Pro-Lab Diagnostics, Richmond Hill, Canada) until further use.

### 2.2 Phenotypic identification and antimicrobial susceptibility testing

Phenotypic antimicrobial susceptibility testing was performed to determine the resistance profiles of the *E. coli* isolates prior to genomic analyses. Minimum inhibitory concentrations (MICs) were determined by broth microdilution in accordance with CLSI M07 (16). Interpretive categories and quality-control ranges were assigned using CLSI M100 (17) and, where veterinary-specific criteria were available, CLSI VET01S (18). Epidemiological cut-off values were obtained from the EUCAST MIC distribution database (14).

Bacterial strains stored at −80 °C were revived the day prior to testing by inoculating into 3 mL of cation-adjusted Mueller-Hinton broth (CAMHB), followed by incubation at 37 °C for 18–24 h. MICs were determined using 96-well microtiter plates (VWR International, LLC., Debrecen, Hungary). Antimicrobial stock solutions were prepared in accordance with CLSI recommendations. Twofold serial dilutions were prepared in cation-adjusted Mueller–Hinton broth to achieve the final concentration ranges reported in Supplementary Table S6. Each test well was prepared with 90 μL of the corresponding antimicrobial dilution (16).

The antimicrobial agents tested comprised multiple classes relevant to veterinary and human medicine, including amoxicillin, amoxicillin–clavulanic acid, ceftriaxone, cefotaxime, cefotaxime–clavulanic acid, imipenem, aztreonam, doxycycline, tobramycin, spectinomycin, gentamicin, neomycin, florfenicol, enrofloxacin, colistin, and a potentiated sulfonamide consisting of trimethoprim and sulfamethoxazole at a 1:19 ratio.

Bacterial suspensions prepared from the overnight cultures were adjusted to a 0.5 McFarland standard using a nephelometer (Thermo Fisher Scientific, Budapest, Hungary). The standardized suspension was subsequently diluted in CAMHB to obtain a working inoculum of approximately 5 × 10^6^ CFU/mL. Within 15 min of inoculum preparation, 10 μL of the working suspension was added to each well containing 90 μL of the corresponding antimicrobial dilution, yielding a final inoculum of approximately 5 × 10^5^ CFU/mL in a total volume of 100 μL per well. Growth-control wells containing inoculated CAMHB without antimicrobial agent and sterility-control wells containing uninoculated CAMHB were included on each plate. MIC values were read using the Sensititre SWIN automated MIC reader and VIZION system software version 3.4 (Thermo Fisher Scientific, 2024). *E. coli* ATCC 25922 was used as a quality-control strain.

### 2.3 Phenotypic confirmation of extended-spectrum β-lactamase production

Phenotypic confirmation of ESBL production was performed according to CLSI M100 (17). Phenotypic confirmation was based on the comparison of MICs of cefotaxime alone and in combination with clavulanic acid. MIC values for cefotaxime and the cefotaxime-clavulanic acid combination were determined using microdilution plates. In all wells containing the clavulanic acid combination, a constant concentration of 4 μg/mL clavulanic acid was maintained throughout the dilution series. Plates were incubated at 37 °C for 18–24 h prior to evaluation.

A strain was considered ESBL-producing if the MIC of cefotaxime in combination with clavulanic acid was reduced by at least three two-fold dilution steps relative to cefotaxime alone (i.e., an ≥8-fold reduction), in accordance with CLSI interpretive criteria. For example, a cefotaxime MIC decreasing from 8 μg/mL to 1 μg/mL in the presence of clavulanic acid represents an eightfold reduction and therefore fulfills the phenotypic criterion for ESBL production.

The cefotaxime–clavulanate test detects clavulanate-inhibited cefotaxime hydrolysis but does not identify the underlying enzyme. Co-production of AmpC β-lactamases or permeability-related mechanisms may alter the apparent phenotype and contribute to phenotype–genotype discordance.

### 2.4 DNA extraction and whole-genome sequencing

Whole-genome sequencing was performed to enable comprehensive genomic characterization of the selected *E. coli* isolates. Genomic DNA was extracted from bacterial suspensions using the Zymo Quick-DNA Fungal/Bacterial Miniprep Kit (Zymo Research, Irvine, CA, USA), according to the manufacturer’s instructions. Mechanical lysis was performed using a TissueLyser LT (Qiagen GmbH, Hilden, Germany) at 50 Hz for 5 min. The extracted DNA was stored at −20 °C until further processing.

Sequencing libraries were prepared using Illumina-compatible chemistry and subsequently converted for sequencing on an MGI platform due to a technical platform change during the study period.

For library preparation, 50 ng of genomic DNA was processed using the TruePrep DNA Library Prep Kit V2 for Illumina (TD501-01, Vazyme Biotech, Nanjing, China). Indexing was performed using Nextera XT Index Kit v2 Sets A and B (Illumina, San Diego, CA, United States). Library preparation steps followed the manufacturer’s protocol, including tagmentation, amplification, and purification. Library concentrations were quantified using the Qubit dsDNA HS Assay Kit (Thermo Fisher Scientific, Waltham, MA, United States).

Illumina-compatible libraries were converted to MGI-compatible format using the MGIEasy Universal Library Conversion Kit (App-A) (MGI Tech, Wuhan, China) according to the manufacturer’s instructions. Single-stranded circular DNA libraries were generated and processed for DNA nanoball (DNB) formation using standard MGI workflows. Library quality and concentration were assessed using Qubit ssDNA HS Assay Kit prior to sequencing.

Final sequencing was performed on an MGI DNBSEQ-G400RS platform using paired-end 150 bp reads (PE150). Sequencing generated an average genome coverage of approximately 80–120 × per isolate.

### 2.5 Genome assembly, quality control and genomic characterization

Raw sequencing data were quality-checked using FastQC v0.11.9, fastp v0.23.2–3, and Bloocoo v1.0.7 to assess read quality, base composition, and adapter contamination (19, 20). High-quality reads were *de novo* assembled using MEGAHIT v1.2.9 and SPAdes v4.0.0, and the resulting assemblies were integrated with GAM-NGS v1.1b to improve continuity and completeness (21–23). Assembly quality was evaluated using QUAST v5.2 and BUSCO v5 (24, 25). GenomeScope v2.2 was used to estimate genome size, sequencing coverage, and k-mer distribution (26). Antimicrobial resistance genes were identified using Resistance Gene Identifier (RGI) v5.1.0 and ABRicate against the Comprehensive Antibiotic Resistance Database (CARD) (27). In this context, sequence identity refers to nucleotide similarity, whereas coverage denotes the proportion of the reference gene length covered by the query sequence.

Assemblies were annotated using Prokka v1.14.6 with lineage-specific settings (−-genus Escherichia --usegenus) (28). Acquired antimicrobial-resistance genes and resistance-associated alleles were additionally identified using NCBI AMRFinderPlus v4.2.7 (29). β-Lactamase determinants were reported at allele level and classified according to their curated functional annotation; thus, TEM, SHV and OXA family membership alone was not considered evidence of an ESBL phenotype. Multilocus sequence types were assigned using the mlst pipeline v2.35.0 against the PubMLST Achtman seven-locus *Escherichia coli* scheme (30). Only complete and unambiguous seven-locus profiles were reported as valid sequence types. Isolates without an assigned ST were subsequently re-examined specifically for the seven Achtman loci (*adk, fumC, gyrB, icd, mdh, purA* and *recA*) using the available sequence data to determine whether a complete profile could be recovered. This targeted reassessment did not yield any additional complete and unambiguous seven-locus profiles. Consequently, isolates for which a definitive ST remained unavailable were retained as unresolved rather than being assigned an inferred or partial sequence type. Phylogroup assignment was additionally performed in silico from the final quality-controlled genome assemblies using EzClermont v1.0.0, which implements the Clermont phylogrouping scheme for whole-genome assemblies and includes supplementary reactions for resolving phylogroups C, E and G. The default minimum contig-length threshold of 500 bp was applied, with partial primer-site detection retained for fragmented draft assemblies. Isolates yielding unresolved, cryptic or internal-control-failure profiles were retained as unresolved rather than being forced into a canonical phylogroup assignment (31, 32).

Predicted plasmid reconstruction and typing were performed using MOB-suite v3.1.9 (33). Reconstructed plasmid bins were characterized according to replicon type, relaxase family, mating-pair formation system and predicted mobility class. β-Lactamase loci identified by AMRFinderPlus were linked to Prokka-annotated contigs and subsequently mapped to MOB-suite chromosome or plasmid bins using the corresponding contig and protein identifiers. Associations were classified as chromosome-predicted, plasmid-predicted or unresolved. Because the analysis was based on short-read draft assemblies rather than complete closed plasmids, all plasmid assignments are reported as bioinformatically predicted associations.

The core genome was identified using Panaroo v1.6.0 in strict cleaning mode (34). To account for the effect of homologous recombination on phylogenetic reconstruction, the resulting core-genome alignment was analyzed using Gubbins v3.4.3. Gubbins iterations used RAxML v8.2.12 under the GTRGAMMA model and generated a recombination-filtered alignment of polymorphic sites (35, 36). This alignment was subsequently analyzed with IQ-TREE v3.0.1 under the GTR + F + G4 substitution model, with branch support evaluated using 1,000 ultrafast bootstrap replicates (37). The final analysis included the 87 study isolates and eight publicly available chicken-associated comparator genomes.

Taxonomic classification and assembly quality were evaluated using Kraken 2 v2.1.3 and CheckM v1.2.2, respectively (38, 39). Virulence-associated loci were screened using ABRicate against VFDB and cross-referenced against matching virulence-associated gene symbols in the final Prokka annotations (40). For isolate-level prevalence summaries, a locus was considered detected when supported by either output, while the underlying evidence source (VFDB, Prokka or both) was retained in Supplementary Table S5. Functional groupings were used descriptively, and detection of one or more constituent loci was not interpreted as evidence for the presence of a complete or functionally expressed virulence system.

For phylogenomic contextualization, publicly available chicken-associated *Escherichia coli* genomes were retrieved from the Bacterial and Viral Bioinformatics Resource Center (BV-BRC) and selected as external comparator genomes using predefined metadata filters, including host species, taxonomic assignment to *E. coli*, complete genome status, and availability of relevant associated metadata such as isolation source and country of origin. The final comparator set was designed to capture phylogenetic diversity across poultry-associated and avian pathogenic *E. coli* lineages, and all included BV-BRC genome identifiers are listed in Supplementary Table S1. Phylogenetic trees were visualized and annotated using iTOL v7.5.1 (41).

Isolate-level assembly metrics, including assembly length, GC content, contig number, N50, L50 and minimum and maximum contig lengths, are reported in Supplementary Table S2.

### 2.6 Statistical analysis and data visualization

Statistical analyses were primarily descriptive and exploratory, and no *a priori* power calculation was performed. Associations of phenotypic ESBL status with production type and geographic region were evaluated using exact contingency-table tests. Accessory-genome composition was evaluated using Jaccard distances and PERMANOVA with 999 permutations, with phenotypic ESBL status as the explanatory variable. Anatomical source was incorporated in the graphical annotation but was not included as an additional explanatory factor. Statistical analyses were performed in R v4.5.3, and two-sided *p* values below 0.05 were considered statistically significant.

Multidrug resistance (MDR) was defined as non-susceptibility to at least one agent in three or more antimicrobial categories. XDR and PDR categories were not assigned because the tested panel did not include the complete agent sets required for standard international category-level classification.

## 3 Results

### 3.1 Composition of the analytical isolate collection

*Escherichia coli* was recovered from 241/345 cloacal swabs (69.9%) and 169/345 tracheal swabs (49.0%). These recovery frequencies are presented descriptively because comparison between the two paired anatomical sampling sites was not a prespecified analytical objective of the study. The final analytical collection comprised 87 chicken-derived *E. coli* isolates. Sixty-two isolates originated from cloacal samples and 25 from tracheal samples. According to production type, 49 isolates originated from laying flocks, 23 from broiler flocks and 15 from breeding flocks. The isolates represented seven Hungarian regions: Northern Hungary (*n* = 24), Central Transdanubia (*n* = 20), Southern Transdanubia (*n* = 14), Central Hungary (*n* = 9), Northern Great Plain (*n* = 8), Western Transdanubia (*n* = 8) and Southern Great Plain (*n* = 4). These distributions describe the screened analytical collection and were not used to infer regional or production-level prevalence.

### 3.2 Phenotypic antimicrobial susceptibility results

Because the final analytical set consisted of isolates selected on the basis of initial phenotypic profiles suggestive of potential ESBL production, the resistance frequencies reported below describe this phenotypically enriched study collection rather than prevalence in the underlying farm population.

The complete MIC distributions for all tested β-lactam and non-β-lactam antimicrobial agents are provided in Supplementary Table S6. Overall susceptibility categories are summarized in Figure 1. The highest resistance frequencies were observed for trimethoprim–sulfamethoxazole (89.7%), amoxicillin (67.8%), neomycin (67.8%), gentamicin (57.5%), florfenicol (57.5%), tobramycin (56.3%) and enrofloxacin (55.2%).

**Figure 1:**
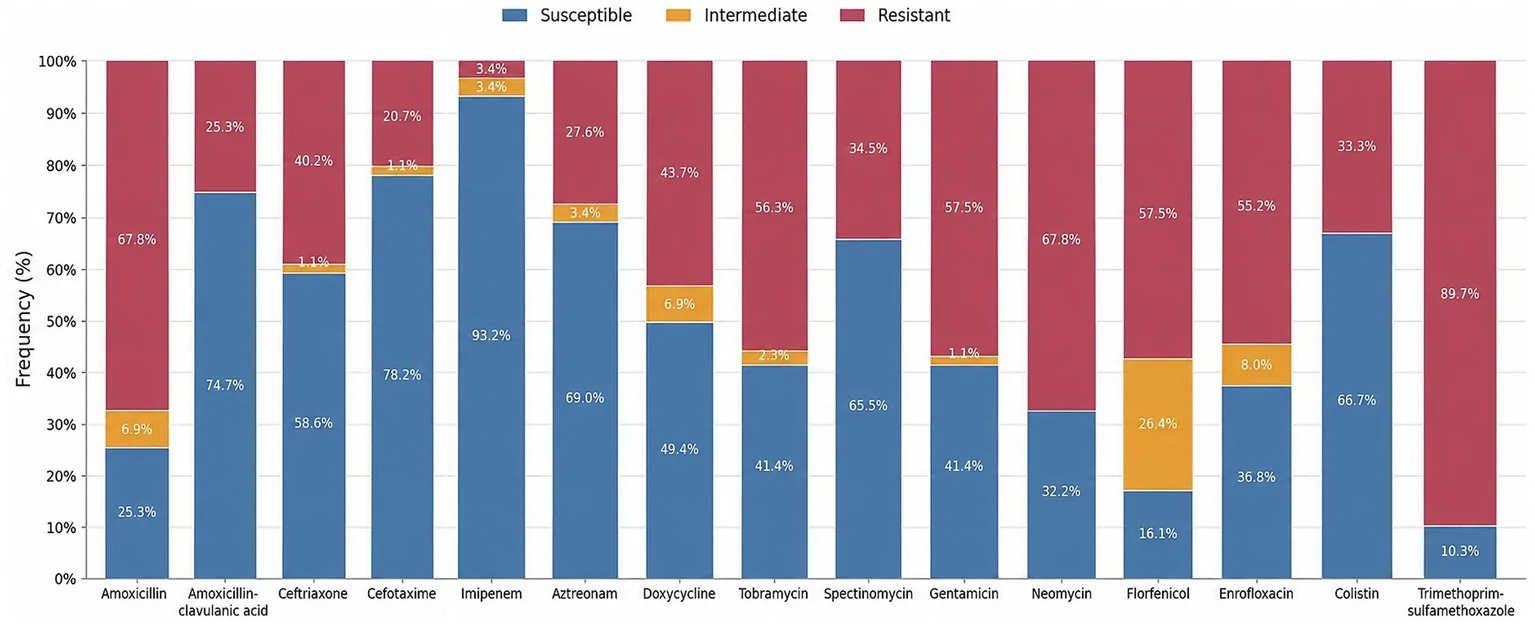
Antimicrobial susceptibility categories of 87 chicken-derived *Escherichia coli* isolates for compounds with defined interpretive breakpoints. Bars show the percentages of isolates classified as susceptible, intermediate and resistant. Percentages were calculated from all 87 isolates for each antimicrobial agent shown.

### 3.3 Phenotypic ESBL status across sample and production categories

Based on the predefined criterion of an at least eightfold reduction in the cefotaxime MIC in the presence of clavulanic acid, 19/87 isolates (21.8%) were classified as phenotypically ESBL-positive. Phenotypic ESBL positivity was detected in 15/62 cloacal isolates (24.2%) and 4/25 tracheal isolates (16.0%). These source-specific proportions are reported descriptively, as anatomical-site comparison was not a prespecified objective of the study. ESBL positivity was identified in 13/49 isolates from laying flocks (26.5%), 5/23 from broiler flocks (21.7%) and 1/15 from breeding flocks (6.7%); phenotypic ESBL status was not associated with production type (Fisher’s exact test, *p* = 0.290). Positive isolates were distributed across several geographic regions, without evidence of concentration in a single production category or sampling source. Phenotypically ESBL-positive isolates occurred in all but one geographic region, and ESBL status was not significantly associated with region (Fisher’s exact test, *p* = 0.530).

### 3.4 Multidrug resistance categories

Using the tested antimicrobial panel, 84/87 isolates (96.6%) met the MDR criterion. Non-susceptibility involved as many as 15 of the 16 tested compounds, indicating that β-lactam-associated resistance frequently occurred within a broader multidrug-resistance background.

### 3.5 Non-β-lactam resistance determinants

Non-β-lactam antimicrobial-resistance determinants were detected in 54/87 isolates (62.1%). Quinolone-resistance determinants were the most widely distributed, with *qnr*-family genes detected in 32/87 isolates (36.8%); *qnrB*-family determinants predominated (26/87, 29.9%), comprising *qnrB* in 25 isolates and *qnrB1* in one isolate. Tetracycline-resistance genes were identified in 24/87 isolates (27.6%), including *tet(A)* in 9/87 (10.3%), *tet(J)* in 9/87 (10.3%) and *tet(B)* in 8/87 (9.2%). Aminoglycoside-modifying enzyme genes occurred in 18/87 isolates (20.7%), with *aph(6)-Id* (7/87, 8.0%), *aadA1* (6/87, 6.9%) and *aph(3″)-Ib* (6/87, 6.9%) among the most frequent determinants. Folate-pathway resistance determinants of the sul and/or dfrA families were present in 23/87 isolates (26.4%); *sul*-family genes and *dfrA*-family genes were each detected in 14/87 isolates (16.1%). Phenicol-resistance determinants were identified in 15/87 isolates (17.2%), most commonly *catA* (9/87, 10.3%) and *floR* (5/87, 5.7%). No known plasmid-mediated *mcr* determinant was detected. Because multiple resistance genes frequently co-occurred within individual isolates, the reported determinant categories are not mutually exclusive. Complete isolate-level resistance-gene profiles are provided in Supplementary Table S3.

### 3.6 Allele-level distribution and predicted genomic context of β-lactamase genes

Allele-level AMRFinderPlus analysis identified 62 β-lactamase-associated gene calls in 54/87 isolates. The principal ESBL determinants were *bla*CTX-M-1, detected in 11 isolates (12.6%), and *bla*_CTX-M-15_, detected in three isolates (3.4%). The acquired AmpC β-lactamase *bla*_CMY-2_ was identified in four isolates (4.6%), and one additional isolate carried an ACT-family class C β-lactamase.

The most frequent non-ESBL β-lactamase allele was *bla*_TEM-1_ (30 isolates), followed by *bla*_TEM-135_ (five isolates), *bla*_TEM-32_ (two isolates) and *bla*_TEM-30_ (one isolate). Three additional TEM-family detections could not be resolved to a specific allele. Single occurrences of *bla*_SHV-11_ and an OXA-1-family β-lactamase were also identified. TEM-1, TEM-135 and SHV-11 were interpreted as broad-spectrum rather than extended-spectrum β-lactamases, whereas TEM-30 and TEM-32 were classified as inhibitor-resistant TEM variants.

Among the 19 phenotypically ESBL-positive isolates, 12 (63.2%) carried a *bla*_CTX-M_ allele. Conversely, 12/14 *bla*_CTX-M_-positive isolates (85.7%) fulfilled the phenotypic ESBL criterion. Seven phenotypically positive isolates lacked a detected CTX-M allele, whereas two CTX-M-positive isolates did not fulfill the phenotypic confirmation criterion. These discrepancies may reflect differences in gene expression, additional β-lactamase backgrounds, permeability-related mechanisms or technical variation in phenotypic expression (Figure 2).

**Figure 2:**
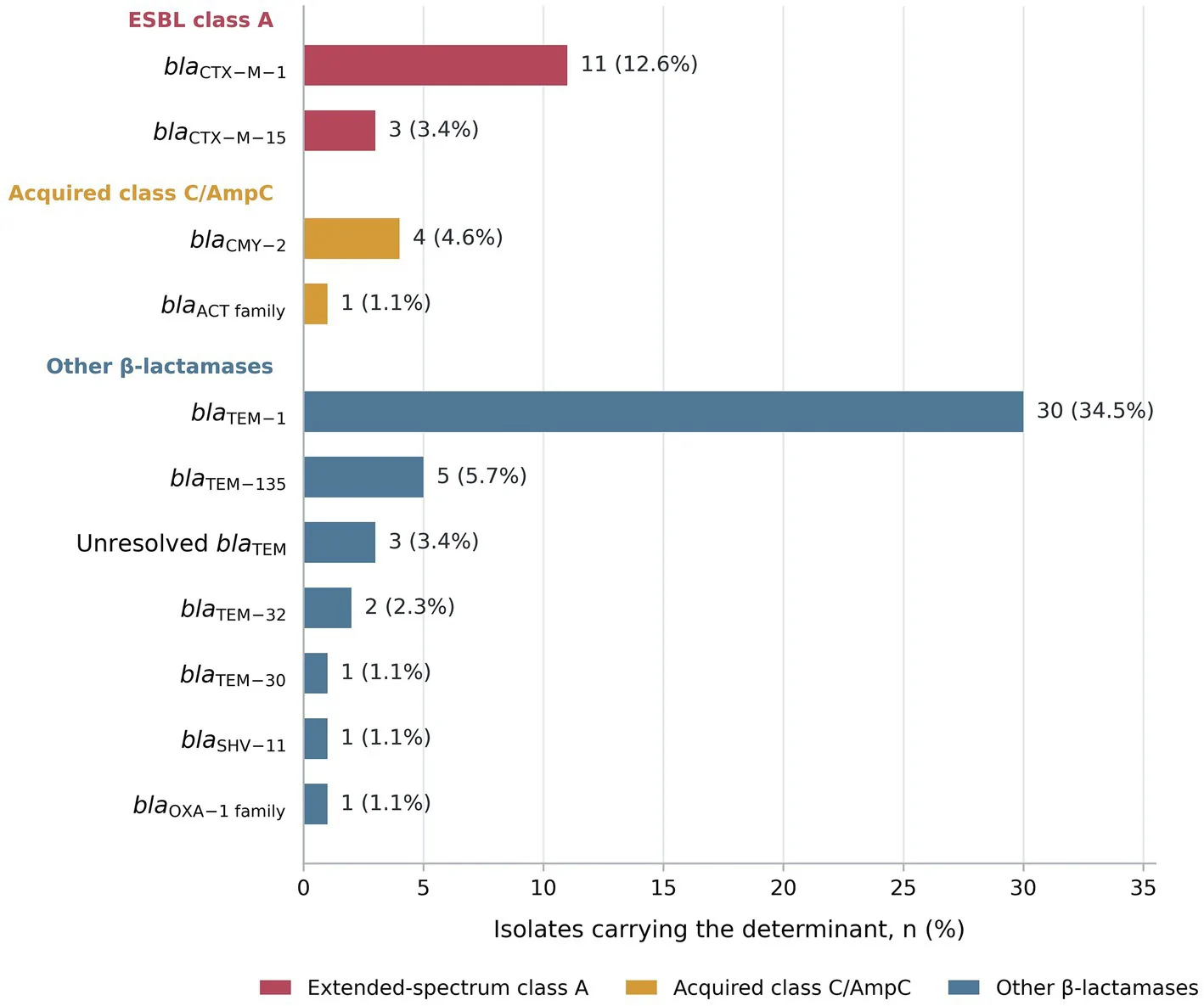
Allele-level distribution of β-lactamase determinants among 87 chicken-derived *Escherichia coli* isolates. Bars show the number and percentage of isolates carrying each determinant. *bla*_CTX-M-_1 and *bla*_CTX-M-15_ encode extended-spectrum class A β-lactamases, whereas *bla*CMY-2 and the ACT-family determinant encode acquired class C/AmpC β-lactamases. TEM, SHV and OXA-family determinants were interpreted according to their allele-level AMRFinderPlus annotation and were not collectively classified as ESBLs.

Three of the 87 isolates (3.4%) exhibited an imipenem MIC of 4 μg/mL. No acquired carbapenemase gene was detected in these isolates. Their genomic backgrounds included combinations of CTX-M, CMY, TEM, SHV or OXA-family β-lactamases, but the current dataset did not permit definitive assignment of the reduced imipenem susceptibility to a specific mechanism (Figure 3).

**Figure 3:**
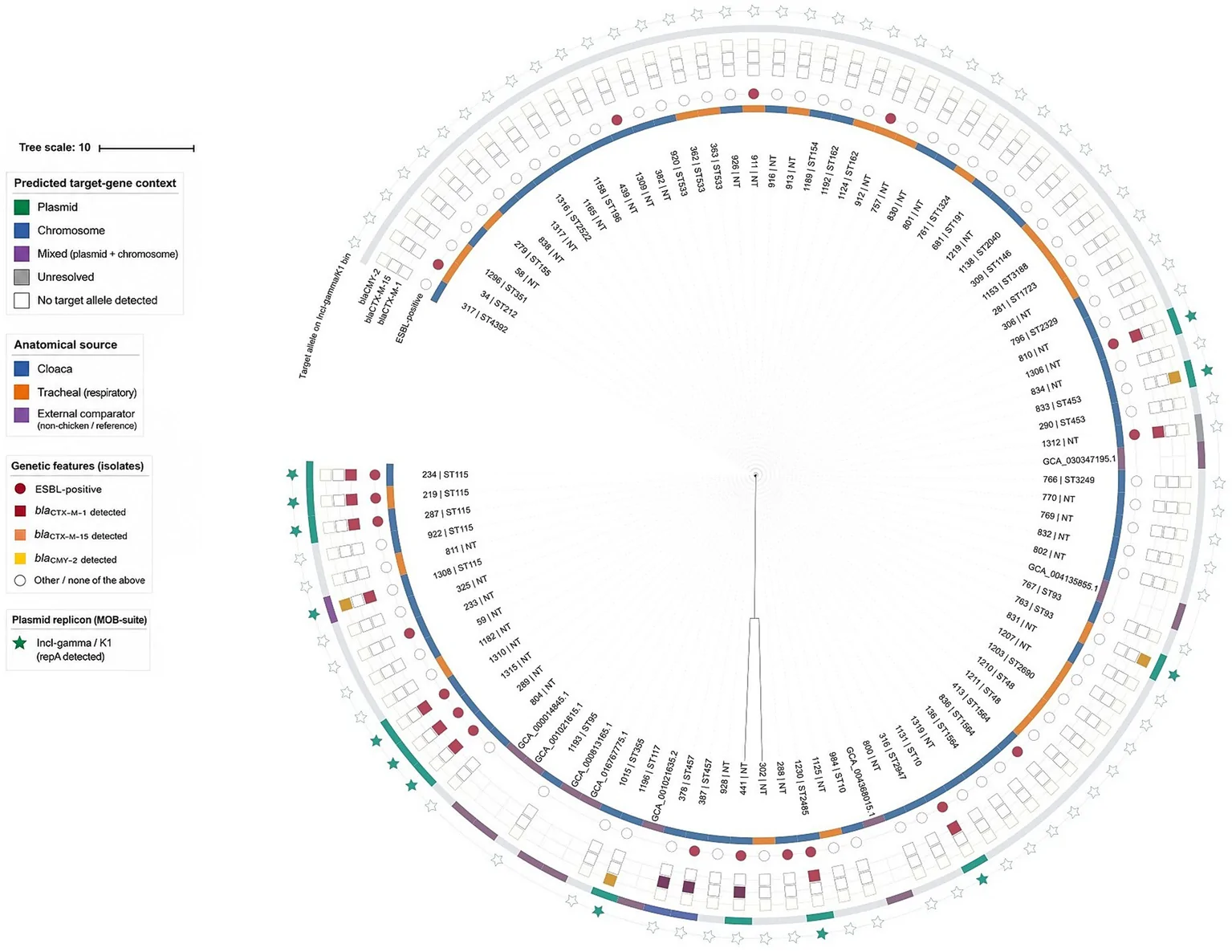
Circular recombination-filtered core-genome single-nucleotide polymorphism phylogeny of 87 chicken-derived *Escherichia coli* isolates from Hungary and eight external chicken-associated comparator genomes. Homologous recombination was identified and removed using Gubbins, and the maximum-likelihood tree was reconstructed from 77,508 filtered polymorphic sites using IQ-TREE under the GTR + F + G4 model with 1,000 ultrafast bootstrap replicates. From inner to outer tracks, annotations indicate anatomical source, phenotypic ESBL status, *bla*CTX-M-1, *bla*CTX-M-15 and *bla*CMY-2 carriage, predicted target-gene context, and association with an IncI-gamma/K1 plasmid bin. Study-isolate labels include sequence type where available; NT denotes isolates for which no complete Achtman ST was assigned in the present analysis. External comparator genomes were included for phylogenetic contextualization and were not assigned study-specific phenotypic or plasmid-context annotations. Branch-support values are omitted from the circular graphical display for clarity; the complete IQ-TREE Newick file containing node-support values is provided as Supplementary Data S1. Plasmid assignments represent MOB-suite predictions derived from short-read draft assemblies.

### 3.7 Sequence-type diversity and recombination-filtered phylogeny

A complete Achtman multilocus sequence type was assigned to 44/87 isolates, representing 30 distinct sequence types. ST115 was the most frequent sequence type but comprised only five isolates. ST1564 and ST533 each included three isolates, whereas ST10, ST48, ST93, ST162, ST453 and ST457 each occurred twice. The remaining 21 assigned sequence types were represented by single isolates. Forty-three isolates did not receive an assigned Achtman ST in the assembly-based analysis. Targeted re-examination of the seven Achtman loci in these isolates did not recover any additional complete and unambiguous profiles.

Complementary in silico phylogroup assignment resolved 75/87 isolates (86.2%) into one of the major *E. coli* phylogroups. Phylogroup B1 was the most frequent (29/87, 33.3%), followed by A (20/87, 23.0%) and D (15/87, 17.2%). Less frequent phylogroups included B2 (3/87, 3.4%), E (3/87, 3.4%), C (2/87, 2.3%), F (2/87, 2.3%) and G (1/87, 1.1%). Twelve isolates (12/87, 13.8%) yielded unresolved or non-canonical Clermont profiles and were therefore not assigned to a major phylogroup. The broad distribution across multiple phylogroups provided an additional, lower-resolution measure of the phylogenetic heterogeneity observed by MLST and core-genome analysis.

Gubbins analysis of the Panaroo core-genome alignment generated a recombination-filtered alignment containing 77,508 polymorphic sites across the 87 study isolates and eight external chicken-associated comparator genomes. The resulting IQ-TREE phylogeny resolved multiple deeply separated lineages, consistent with the high sequence-type diversity of the collection. Phenotypically ESBL-positive and *bla*_CTX-M_-positive isolates occurred on several distinct branches and were not confined to ST115 or any other single sequence type. Some closely related isolates formed small local clusters, but the overall topology did not support expansion of one dominant ESBL-associated clone.

Cloacal and tracheal isolates were interspersed across the phylogeny, with no obvious source-specific clustering. The external comparator genomes also occupied multiple positions within the tree, placing the Hungarian isolates within a broader and heterogeneous chicken-associated *E. coli* genomic background.

### 3.8 Predicted plasmid content and β-lactamase-associated plasmid backgrounds

MOB-suite results were available for 86/87 isolates. The genomic context of the *bla*_CTX-M-1_ determinant in isolate 1,312 remained unresolved. Among the 86 isolates with available MOB-suite results, the most frequently detected replicon families were IncFIB (65/86, 75.6%), IncFIC (35/86, 40.7%), IncFIA (34/86, 39.5%), IncI-gamma/K1 (20/86, 23.3%), IncHI1B (19/86, 22.1%) and IncFII (18/86, 20.9%).

Of the 62 β-lactamase-associated gene calls, 46 (74.2%) were assigned to predicted plasmid bins, 15 (24.2%) to chromosome-predicted sequences and one (1.6%) remained unresolved. Among the 46 plasmid-associated calls, 32 occurred in plasmid bins predicted to be conjugative, seven in mobilizable bins and seven in non-mobilizable bins.

Ten of the 11 *bla*_CTX-M-1_ occurrences were computationally assigned by MOB-suite to predicted conjugative IncI-gamma/K1 plasmid bins, while the remaining occurrence was unresolved. Of the three *bla*_CTX-M-15_ genes, one was assigned to a predicted conjugative IncFIA/IncFII plasmid bin and two to chromosome-predicted sequences. Three of the four *bla*_CMY-2_ genes were computationally assigned to predicted conjugative IncI-gamma/K1 plasmid bins, whereas one was chromosome-predicted. These results indicate a recurrent predicted association of *bla*_CTX-M-1_ and *bla*_CMY-2_ with IncI-gamma/K1 backgrounds, whereas *bla*_CTX-M-15_ occurred in more heterogeneous predicted genomic contexts.

All plasmid-context assignments were computational predictions derived from short-read draft assemblies. They should not be interpreted as experimental confirmation of physical plasmid localization, complete plasmid structure, conjugative transfer, or dissemination. Complete plasmid-bin characteristics and allele-level predicted genomic-context assignments are provided in Supplementary Table S4.

### 3.9 Virulence-associated gene repertoire

Virulence-associated loci showed a heterogeneous distribution across the 87-isolate collection. At least one enterobactin-associated locus was detected in 86/87 isolates (98.9%), at least one curli-associated locus in 85/87 (97.7%), at least one type 1 fimbrial locus in 77/87 (88.5%), and at least one general secretion pathway locus in 77/87 (88.5%). Among more variably distributed determinants, at least one *pap*-family P-fimbrial locus was detected in 54/87 isolates (62.1%). Aerobactin-associated *iuc/iut* loci were identified in 49/87 isolates (56.3%), including *iutA* in 37/87 (42.5%). Capsule-associated *kps* loci occurred in 32/87 isolates (36.8%), while salmochelin-associated *iro* loci were detected in 26/87 (29.9%), including *iroN* in 20/87 (23.0%). Yersiniabactin-associated loci were present in 24/87 isolates (27.6%), with *fyuA* likewise detected in 24/87 (27.6%). The invasion-associated marker *ibeA* was comparatively uncommon, occurring in 4/87 isolates (4.6%). The virulence-associated marker *astA* was detected in 68/87 isolates (78.2%). These group-level frequencies indicate detection of at least one constituent locus and should not be interpreted as evidence that the corresponding operon or functional system was complete or expressed. Because individual virulence-associated markers can occur in both commensal and extraintestinal pathogenic *E. coli* backgrounds, the isolates were not assigned to a clinical pathotype solely on the basis of individual gene detections. Complete isolate-level presence–absence data and the corresponding evidence sources are provided in Supplementary Table S5.

### 3.10 Pan-genome and accessory-genome composition

Pan-genome analysis across the 95-genome phylogenomic dataset identified 22,248 gene clusters, including 517 soft-core, 5,778 shell and 15,953 cloud clusters; no clusters met the 99–100% strict-core threshold. This distribution indicates substantial variation in recovered gene content, although its interpretation must also account for incomplete gene recovery and annotation differences among draft assemblies. Among the 87 study isolates, phenotypic ESBL status was associated with accessory-gene composition (PERMANOVA on Jaccard distances: *F* = 2.26, *p* = 0.005), although substantial overlap remained between the groups (Supplementary Figure S1). This result is interpreted as a collection-level compositional association rather than evidence for a discrete ESBL-specific accessory module.

## 4 Discussion

The phenotypic antimicrobial susceptibility profiles observed among *E. coli* isolates from chickens reveal a high overall burden of antimicrobial resistance across multiple antibiotic classes. Resistance was not restricted to β-lactam compounds but extended to several non–β-lactam agents commonly used in veterinary practice, indicating a broad resistance phenotype within the studied population. Such patterns are consistent with the role of commensal *E. coli* as a sentinel organism for antimicrobial resistance surveillance in food-producing animals.

The observed distribution of minimum inhibitory concentrations, interpreted using both clinical breakpoints and ECOFF values, highlights the coexistence of clinically resistant and non–wild–type subpopulations within the same production system. This dual interpretation provides complementary insights, as clinical breakpoints reflect therapeutic relevance, while ECOFF-based assessments offer information on resistance emergence and population-level shifts.

Taken together, the phenotypic data suggest that the investigated *E. coli* population harbors multidrug resistance traits with potential implications for antimicrobial efficacy, animal health management, and the dissemination of resistance determinants along the food chain. These findings provide the framework for a more detailed discussion of β-lactam resistance patterns and associated extended-spectrum β-lactamase phenotypes, as well as co-resistance to non–β-lactam antimicrobials, which are addressed in the following sections.

Within the screened collection, elevated cefotaxime MICs and clavulanate-related MIC reductions were consistent with ESBL-associated resistance in a subset of isolates. Phenotypic ESBL positivity and *bla*_CTX-M_ carriage overlapped in 12 isolates: 12/19 phenotypically ESBL-positive isolates (63.2%) carried a detected *bla*_CTX-M_ allele, while 12/14 *bla*_CTX-M_-positive isolates (85.7%) fulfilled the phenotypic confirmation criterion. Seven phenotypically positive isolates lacked a detected *bla*_CTX-M_ allele, and two *bla*_CTX-M_-positive isolates did not meet the phenotypic criterion. These findings indicate that the two classifications were related but not interchangeable. Alternative β-lactamase backgrounds, regulatory mechanisms or permeability-associated changes may have contributed to the discordant results.

Because the present dataset was enriched through β-lactam/ESBL-oriented screening, these frequency estimates should be interpreted as characteristics of the screened analytical collection rather than direct prevalence estimates for the underlying flock population. Comparisons with published prevalence studies should therefore be made cautiously.

In the screened analytical collection, all 87 isolates had evaluable cefotaxime results. Eighteen isolates (18/87, 20.7%) were resistant to cefotaxime, and 19/87 (21.8%) fulfilled the predefined phenotypic ESBL criterion. Because isolate inclusion was based on initial phenotypic profiles suggestive of potential ESBL production, these proportions should not be interpreted as flock-level prevalence estimates or compared directly with unselected surveillance studies. Published poultry-associated investigations nevertheless demonstrate substantial variation in cefotaxime resistance and ESBL occurrence across sampling frameworks, production systems and geographic regions (42–55).

Beyond β-lactam resistance, a pronounced multidrug-resistant phenotype was observed among the investigated isolates. Resistance to a broad spectrum of antimicrobial agents was common, with individual isolates exhibiting resistance to up to 15/16 different compounds.

The high frequency of co-resistance indicates substantial co-occurrence of resistance phenotypes. Because farm-level antimicrobial-use data were not available, the present study cannot attribute these patterns to specific antimicrobial selection pressures. Such multidrug resistance profiles in chicken-derived *E. coli* are of particular concern from a One Health perspective, as these bacteria may act as reservoirs for resistance determinants transferable across ecological and host boundaries. Recent veterinary studies have likewise documented substantial antimicrobial-resistance burdens in Hungarian commercial chicken production (56), the potential role of wild birds in environmental AMR dissemination (57), resistome diversity in layer-derived *E. coli* (58), resistance genes and mobile genetic elements in *E. coli* from healthy farm animals (59), and ESBL-associated resistance in chicken-originated *Proteus mirabilis* (60).

The principal detected ESBL genes were *bla*_CTX-M-1_ and *bla*_CTX-M-15_, identified in 11 and three isolates, respectively. By contrast, the frequent TEM-family gene detections were dominated by *bla*_TEM-1_ and *bla*_TEM-135_, which encode broad-spectrum rather than extended-spectrum β-lactamases, together with *bla*TEM-30 and *bla*TEM-32, which encode inhibitor-resistant TEM variants. Family-level grouping of all *bla*_TEM_, *bla*_SHV_ or *bla*_OXA_ determinants as ESBL genes would therefore overestimate the genomic ESBL burden. The detection of *bla*_CMY-2_ in four isolates additionally demonstrates the contribution of acquired AmpC-mediated resistance within the screened collection.

Several mechanisms could contribute to the observed phenotype–genotype discordance. For phenotypically ESBL-positive isolates without a detected *bla*_CTX-M_ allele, possibilities include non-CTX-M ESBL determinants, incomplete recovery of β-lactamase loci in draft assemblies, and variation in phenotypic confirmation. A missed determinant should be evaluated against the actual database content, sequence coverage and reporting thresholds rather than inferred solely from the absence of a *bla*_CTX-M_ hit. Mutations in the chromosomal *ampC* promoter or attenuator region may increase AmpC expression and contribute to cephalosporin resistance; however, AmpC hyperproduction alone is not a sufficient mechanistic explanation for a clavulanate-inhibited ESBL phenotype. AmpC-associated backgrounds can also complicate phenotypic ESBL detection, including masking co-produced ESBLs. Conversely, *bla*_CTX-M_ carriage without phenotypic confirmation may reflect expression-related or assay-related differences (61, 62). The reported analyses do not establish which mechanism applies to the discordant isolates, and targeted sequence review and repeat phenotypic confirmation would be required to distinguish these possibilities.

The three isolates with an imipenem MIC of 4 μg/mL require separate consideration because no acquired carbapenemase gene was detected in their assemblies. Reduced outer-membrane permeability, including loss or downregulation of OmpF and OmpC, may act together with β-lactamase production to reduce carbapenem susceptibility. In *E. coli*, combined CMY-2 production and porin deficiency has been experimentally associated with carbapenem resistance. Nevertheless, the presence of β-lactamase genes in the present isolates does not establish their expression levels or demonstrate a permeability defect (63). The available results therefore identify a phenotype requiring further characterization, rather than a confirmed carbapenemase-mediated mechanism. Repeat susceptibility testing and targeted assessment of porin integrity, expression and carbapenemase activity would be needed for causal attribution.

The predominance of the *bla*_CTX-M-1_ allele over *bla*_CTX-M-15_ is consistent with the recognized importance of *bla*_CTX-M-1_ in European food-animal populations. However, the present analysis adds information beyond gene frequency by demonstrating that most *bla*_CTX-M-1_ determinants were assigned to conjugative IncI-gamma/K1 plasmid bins. The same predicted plasmid background also carried three of four *bla*_CMY-2_ determinants. In contrast, *bla*_CTX-M-15_ occurred in both plasmid-predicted and chromosome-predicted contexts. These differences suggest that individual β-lactamase alleles may follow distinct dissemination routes within poultry-associated *E. coli* populations.

Among the 44 isolates with assigned Achtman sequence types, 30 STs were represented and no individual ST included more than five isolates. However, because complete Achtman STs could not be assigned to 43/87 isolates, MLST alone cannot characterize the full clonal diversity of the collection. The inference that ESBL-associated resistance occurred across multiple bacterial lineages therefore relies primarily on the recombination-filtered core-genome phylogeny, which included all 87 study isolates, with MLST providing complementary lineage information for the typeable subset. Phenotypically ESBL-positive and *bla*_CTX-M_-positive isolates occurred on multiple distinct phylogenetic branches, supporting the presence of ESBL-associated resistance in multiple genomic backgrounds. This interpretation is consistent with previous genomic evidence that cephalosporin-resistance genes can occur across diverse *E. coli* backgrounds (64). However, the present cross-sectional collection does not establish individual plasmid-transfer events or their direction.

The complementary phylogroup analysis further demonstrated that the collection encompassed diverse evolutionary backgrounds. B1 and A predominated and together accounted for 49/87 isolates (56.3%), while D represented a further 15/87 isolates (17.2%); B2, C, E, F and G were less frequent. Although phylogroups A and B1 have historically been associated more frequently with intestinal or commensal *E. coli* populations, whereas B2 and, to a lesser extent, D are often enriched among extraintestinal pathogenic lineages, these ecological associations are neither absolute nor sufficient for pathotype assignment (65). Indeed, substantial phylogroup diversity occurs among fecal *E. coli* populations, and pathogenic potential cannot be inferred reliably from phylogroup membership alone. In the present study, all isolates originated from clinically healthy chickens, and virulence-associated loci were distributed mosaically across multiple genomic backgrounds. We therefore interpret phylogroup membership as a complementary descriptor of population structure rather than as evidence that an individual isolate was either pathogenic or commensal.

The plasmid analysis provides complementary, hypothesis-generating genomic context for this pattern. The recurrent computational assignment of *bla*_CTX-M-1_ to predicted conjugative IncI-gamma/K1 plasmid bins is compatible with a possible contribution of mobile genetic elements to the occurrence of this resistance determinant across distinct bacterial lineages. However, the present short-read data do not experimentally demonstrate plasmid localization, conjugative transfer, or individual dissemination events. Conversely, the heterogeneous predicted contexts of *bla*_CTX-M-15_ indicate that not all ESBL alleles in the collection shared the same predicted genomic context. These observations provide additional genomic context beyond gene-frequency data and core-genome phylogeny but should be interpreted as bioinformatic predictions rather than direct evidence of plasmid-mediated transmission.

Virulence-associated genes were also distributed mosaically across the collection. Adhesion, iron-acquisition, capsule and invasion-associated markers occurred in several genomic backgrounds, but their presence alone does not establish clinical pathogenicity or zoonotic transmission. The virulence findings should therefore be interpreted as genomic potential rather than evidence that individual isolates caused disease or moved between poultry and humans. The resistance and virulence findings must also be distinguished at the level of genomic linkage. Detection of *bla*_CTX-M_ and virulence-associated loci in the same isolate does not establish their location on the same plasmid. Such an association would require explicit mapping of both classes of loci to the same reconstructed plasmid bin and should still be described as predicted rather than structurally confirmed.

The primary-isolation strategy is important when interpreting recovery frequencies. Unlike targeted European surveillance protocols for ESBL-/AmpC-producing *E. coli*, which combine enrichment with cefotaxime-containing selective culture, the present study used a medium without β-lactam selection for initial *E. coli* recovery and applied resistance-oriented selection subsequently. These approaches address different detection objectives: selective procedures are designed to recover resistant subpopulations even when they represent a small fraction of the bacterial population. Consequently, the recovery frequencies and isolate-level resistance proportions reported here should not be compared directly with positivity estimates obtained through targeted selective surveillance (66).

Several limitations should be considered. First, the collection was deliberately enriched through β-lactam/ESBL-oriented screening and was not designed to estimate the prevalence of resistant *E. coli* in Hungarian chicken flocks. Second, cefoxitin, ertapenem and temocillin were not included in the original antimicrobial panel. The phenotypic AmpC interpretation is therefore limited, and the three isolates with an imipenem MIC of 4 μg/mL cannot be characterized as carbapenemase producers; notably, no acquired carbapenemase gene was detected. Finally, MOB-suite results represent predictions derived from short-read draft assemblies and do not provide the same structural resolution as complete hybrid-assembled plasmids. These limitations do not invalidate the observed allele-level, sequence-type or phylogenetic diversity but define the level at which the results should be interpreted.

A substantial limitation of the sequence-type analysis was the absence of an assigned Achtman ST in 43/87 isolates (49.4%). This result should not be interpreted as evidence that all unresolved profiles arose from the same technical cause. Targeted re-examination of the seven Achtman loci was performed for the unresolved isolates, but no additional complete and unambiguous profiles could be recovered. Fragmented or incomplete allele recovery may contribute, but divergent alleles, ambiguous calls and profiles not represented in the applied database must also be considered. The reported genome-wide sequencing coverage does not resolve coverage or sequence ambiguity at individual MLST loci. Consequently, the observed ST distribution describes the 44 typeable isolates and may not capture the full lineage diversity of the collection.

## 5 Conclusion

In conclusion, ESBL- and AmpC-associated resistance in this screened chicken-derived *E. coli* collection occurred across multiple core-genome phylogenetic lineages rather than being confined to a single dominant lineage. Among the 44 isolates with assigned Achtman STs, 30 distinct STs were identified, although the absence of complete ST assignments for 43/87 isolates limits conclusions based specifically on MLST. The *bla*_CTX-M-1_ allele was frequently assigned by MOB-suite to predicted conjugative IncI-gamma/K1 plasmid bins; this computational association is compatible with, but does not experimentally demonstrate, a contribution of mobile genetic elements to its distribution across different genomic backgrounds. These findings support continued genomic surveillance of poultry-associated *E. coli*, while direct assessment of plasmid transfer and cross-sector transmission will require approaches providing experimental or higher-resolution structural evidence together with contemporaneous animal, food, environmental and human datasets.

## Data Availability

Sequencing data have been deposited in NCBI under BioProject PRJNA1459470. Isolate-level assembly metrics, antimicrobial phenotypes, MLST profiles, phylogroup assignments, antimicrobial-resistance determinants, predicted plasmid contexts, virulence-associated gene profiles and MIC distributions are provided in Supplementary Tables S2–S6. The complete maximum-likelihoodphylogeny, including IQ-TREE node-support values, is provided in Newick format as Supplementary Data S1.
