# Supplementary material for: Polyclonal distribution and predicted plasmid context of ESBL- and AmpC-associated resistance in chicken-derived Escherichia coli from Hungary
Source: Front Vet Sci. 2026 Oct 2;13:1959409. doi: 10.3389/fvets.2026.1959409 (PMC13673037; doi:10.3389/fvets.2026.1959409)
Supplement: Supplementary file 1 [file Image_1.pdf]

Supplementary Material

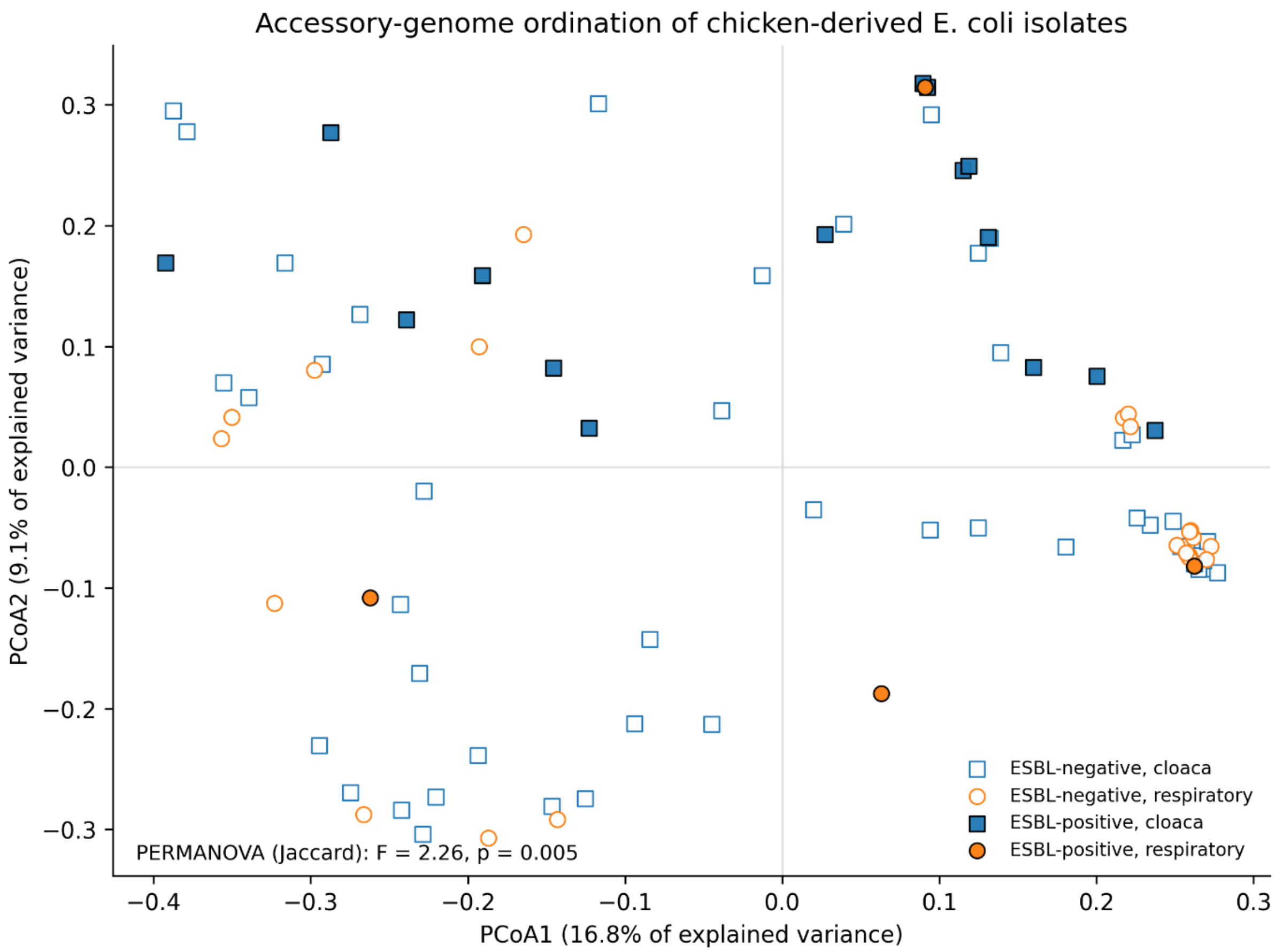

Supplementary Figure S1 Accessory-genome ordination of 87 chicken-derived *Escherichia coli* isolates from Hungary based on gene presence–absence profiles. Principal coordinates analysis (PCoA) was performed using Jaccard distances. Symbols indicate phenotypic ESBL status and anatomical source (cloacal or respiratory). The first two axes explained 16.8% and 9.1% of the total variance, respectively. ESBL-positive and ESBL-negative isolates showed significant separation in accessory-genome composition, although partial overlap remained (PERMANOVA on Jaccard distances: $F = 2.26$, $p = 0.005$).
